# Immunometabolic reprogramming in macrophages infected with active and dormant *Cryptococcus neoformans*: differential modulation of respiration, glycolysis, and fatty acid utilization

**DOI:** 10.1128/iai.00487-24

**Published:** 2024-12-23

**Authors:** Clara Luna Marina, Raffael J. Araújo de Castro, Paula Bellozi, Ana M. Cruz, Pedro Henrique Bürgel, Paul G. Weightman Potter, Craig Beall, Aldo Henrique Tavares, Andreza De Bem, Alexandre Alanio, Carolina Coelho, Anamélia Lorenzetti Bocca

**Affiliations:** 1 Laboratory of Applied Immunology, Institute of Biology Sciences, University of Brasília 223023 , Brasília, Brazil; 2 Laboratory of Bioenergetics and Metabolism, Institute of Biology Sciences, University of Brasília 223023 , Brasília, Brazil; 3 Faculty of Health and Life Sciences, University of Exeter, Exeter, United Kingdom; 4 Graduate Program in Microbial Biology, Department of Cell Biology, Institute of Biological Sciences, Laboratory of Microorganism, Faculty of Ceilândia, University of Brasília 223023 , Brasília, Brazil; 5 Translational Mycology Research Group, National Reference Center for Invasive Mycoses and Antifungals, Mycology Department, Institut Pasteur, Université Paris Cité 555089 , Paris, Île-de-France, France; 6 MRC Centre for Medical Mycology at University of Exeter 601337 , Exeter, Devon, United Kingdom; 7 Bi-Institutional Translational Medicine Platform, Oswaldo Cruz Foundation (Fiocruz) 37903 , Rio de Janeiro, State of Rio de Janeiro, Brazil; NIH, NIAID, Washington DC, USA

**Keywords:** *Cryptococcus neoformans*, VBNC, dormancy, macrophage, immunometabolism, *Fabp4*, *Fabp1*, *Acod1*

## Abstract

Dormancy is an adaptation in which cells reduce their metabolism, transcription, and translation to stay alive under stressful conditions, preserving the capacity to reactivate once the environment reverts to favorable conditions. Dormancy and reactivation of *Cryptococcus neoformans* (*Cn*) are closely linked to intracellular residency within macrophages. Our previous work showed that *in vitro* murine macrophages rely on the viable but not cultivable (VBNC—a dormancy phenotype) fungus from active *Cn*, with striking differences in immunometabolic gene expression. Here, we analyzed the influence of VBNC and active *Cn* on the immunometabolism of infected macrophages, combining metabolic gene expression, mitochondrial membrane potential (ΔΨm), oxygen consumption analysis, and uptake of glucose and fatty acids. The active fungus induced mitochondrial depolarization, and increased glycolysis and mitochondrial oxygen consumption. VBNC infection in bone marrow-derived macrophage (BMDM) caused an attenuated modification in mitochondrial metabolism. However, we found differences in BMDM infected with VBNC vs those infected with active fungus, where VBNC induced an increment in fatty acid uptake in M0 and M1 BMDM, measured by incorporation of BODIPY-palmitate, accompanied by an increase in expression of fatty acid transporters *Fabp1* and *Fabp4*. Overall, distinct fatty acid-related responses induced by VBNC and active *Cn* suggest different immunomodulatory reactions, depending on the microbial growth stage. We posit that, for VBNC, some of these macrophage metabolic responses reflect the establishment of prolonged microbial intracellular residency and possibly initial stages of granuloma formation.

## INTRODUCTION

Cryptococcosis is the most prevalent fungal infection in patients with AIDS, accounting for approximately 19% of AIDS-related deaths globally and causing an estimated 112,000 deaths each year (1). Due to its significant mortality rate, *Cryptococcus neoformans* (*Cn*), the causative agent of cryptococcosis, has been included in the World Health Organization fungal priority pathogen list. Humans and animals are frequently exposed to *Cn*, a ubiquitous environmental yeast, but in healthy individuals, primary infections often go unnoticed or are asymptomatic (2). The disease mainly affects immunocompromised individuals, where the fungus can colonize the central nervous system, leading to meningoencephalitis, with mortality rates reaching 20% even with appropriate treatment (1–5).

In most cases, cryptococcal infections are likely cleared by the immune system of immunocompetent humans (3), although there is evidence of latent, contained infection in some individuals (6). Studies from the 1990s and early 2000s showed that 70% of children older than 5 years exhibit serological reactivity to cryptococcal antigens, and this reactivity remains prevalent in adults (7). This indicates that humans are commonly exposed to *Cn*, even though many do not develop any overt disease or identifiable symptoms (7, 8). The first evidence of dormant cryptococcal infection emerged from the genotypic analysis of strains isolated from patients with imported cryptococcosis. These strains were more similar to those from the patients’ countries of origin than those from their current residence, suggesting that the infection likely occurred during childhood but remained dormant (9, 10). This notion was confirmed by mouse model infection with clinical strains, in which a latent infection was reactivated after the deletion of CD4+ T cells (11). These findings support the hypothesis that many cases of cryptococcosis arise from the reactivation of dormant *Cn* and or those encased in immune granulomas (6, 10–12).

Recently, our research group demonstrated that macrophage exposure can activate viable but not cultivable (VBNC) *Cn* into actively growing culturable cells. Specifically, resting bone marrow-derived macrophage (BMDM—M0) induces the reactivation of VBNC yeast, while M1 pro-inflammatory BMDM induces fewer reactivation events (13). In line with differential responses to dormant *Cn*, we observed that, unlike active yeast, VBNC *Cn* enhanced the production of the chemokine ligand (CCL)4 in macrophages (13). Transcriptomic analysis further revealed that VBNC *Cn* triggered differential expression of metabolic genes, including those in which VBNC induced the expression of genes related to triglyceride sequestering, proton transport, nitric oxide (NO) synthesis, macrophage chemotaxis, cholesterol biosynthesis, and ATP hydrolysis compared to active *Cn* infection (13).

Macrophages are known to dynamically reprogram their metabolism in response to environmental factors, including nutrient availability and inflammatory milieu, and the recognition of pathogen-associated molecular patterns (PAMPs) and damage-associated molecular patterns (14). These metabolic reprogramming shifts are essential for balancing the supply and demand of necessary biomolecules, including ATP, NAD+/NADH, and macromolecules required for inflammation and microbicidal molecules (14). This metabolic reprogramming aligns with the classical M1 and M2 macrophage polarization, although the M1-M2 dichotomy is oversimplified (15).

*In vitro*, M1 macrophages (treated with lipopolysaccharide [LPS] and interferon gamma [IFN-γ]) exhibit high rates of glucose consumption and aerobic glycolysis, fueling pro-inflammatory cytokines and reactive oxygen species (ROS), compared to M0 (unstimulated macrophages). They also engage in fatty acid synthesis, which supports prostaglandin production and membrane remodeling (16, 17). At the same time, M1-macrophages show increased lipid body formation, likely due to a reduced mitochondrial beta-oxidation of exogenous lipids (instead of *de novo* fatty acid synthesis) (18). In contrast, M2 cells demonstrate enhanced oxidative phosphorylation and are associated with anti-inflammatory cytokine production, angiogenesis, and tissue repair processes (19). However, many of these observations are based on single PAMP stimulation, which does not recapitulate the complexity of *in vivo* macrophage-microbe interactions (15, 20), and cell metabolism against infection varies depending on the stimulus received and origin of the macrophage (14).

Building on this understanding, we hypothesized that macrophage responses to VBNC *Cn* versus active *Cn* represent distinct immunometabolic adaptations. Our previous findings pointed to differential production of chemokines and metabolic gene expression profiles in BMDMs exposed to VBNC versus active *Cn*. In this study, we sought to delve deeper into these responses, aiming to elucidate how BMDM metabolically reprograms in the presence of VBNC *Cn*. Through this, we hope to shed light on the mechanisms underlying macrophage responses to dormant fungal cells and their implications for immune defense.

## RESULTS

### VBNC *Cn* causes no detectable changes in mitochondrial membrane potential or ROS production in bone marrow-derived macrophages

Our previous work showed mitochondrial depolarization following infection with active *Cn* both *in vitro* and *in vivo* (21). Additionally, distinct transcriptional profiles emerge in BMDM infected with active or VBNC *Cn* (13). Given the importance of the central role of mitochondria in cellular metabolism and defense, we evaluated the influence of VBNC versus active *Cn* on BMDM mitochondrial membrane potential (ΔΨm) using tetramethylrhodamine methyl ester (TMRM) (21). To mimic the uncultivable state of the fungi, we also tested a mixture of 99% heat-killed (HK) with 1% active fungi (HK + 1). Both naïve BMDM (M0-BMDM) and BMDM pre-stimulated with LPS and IFN-γ (M1-BMDM) were included in our analyses.

**Fig 1:**
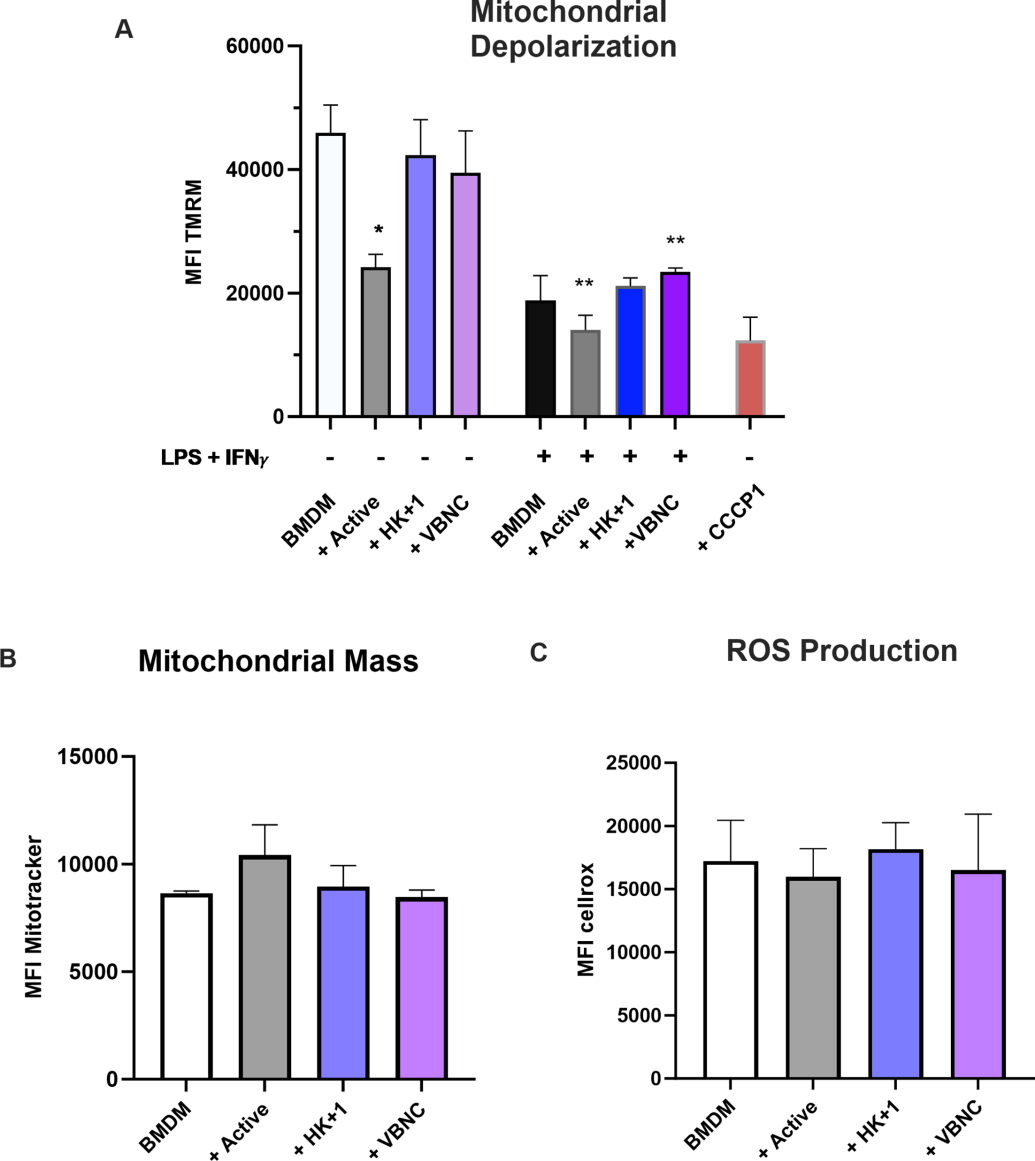
VBNC *Cn* does not induce detectable mitochondrial depolarization in infected BMDM. (A–C) MFI of (**A**) TMRM for depolarization measurement, (**B**) Mitotracker Green for mitochondrial mass measurement, and (**C**) CellRox Green for ROS production measurement in BMDM infected with active, HK + 1% active (HK + 1), or VBNC *Cn*. When specified, cells were also treated with LPS and IFN-γ 24 h (M1-BMDM) before infection and restimulated at the moment of infection; carbonyl cyanide m-chlorophenyl hydrazone 1 (CCCP1) (50 μM) was added 15 minutes before the end of the experiment as a depolarization control. One-way analysis of variance, where *P < 0.05, **P < 0.01, ***P < 0.001, and ****P < 0.0001 compared to the uninfected BMDM group. Figure shows mean and SEM of one representative experiment of three independent experiments with at least three replicates per experimental condition.

As expected, M0-BMDM infected by active *Cn* for 24 h significantly reduced ΔΨm, indicating mitochondrial depolarization, as reflected by the reduction in TMRM median fluorescence intensity (MFI) (Fig. 1A, gating strategy in Fig. S1A), consistent with our previous results (21). In contrast, M0- and M1-BMDM infected with VBNC and HK + 1 had no detectable changes in mitochondrial ΔΨm when compared to non-infected BMDM. Across all fungal infections, we detected no changes in mitochondrial mass (assessed by Mitotracker Green staining) (Fig. 1A and B), supporting the notion that the observed depolarization is not associated with mitochondrial damage, as reported before (21). Notably, we noted depolarization of ΔΨm in M1-BMDM, when compared to M0-BMDM, in line with previous results showing that LPS and IFN-γ treatment can cause both hyper- and depolarization of mitochondria, depending on treatment time and concentration (22).

We also evaluated ROS production using CellRox Green, and no significant differences were observed across any infection conditions (Fig. 1C). This result was expected since the *in vitro* infection of BMDM with *Cn* does not significantly alter the production of ROS (21). These findings highlight that infection with VBNC induces minimal changes in mitochondrial ΔΨm compared to active *Cn* infection, and suggest that *Cn* active fungal growth is required for eliciting mitochondrial depolarization in macrophages. These results support our hypothesis that VBNC-infected macrophages display a different metabolic response compared with active *Cn*-infected BMDM, and correlate with key previously reported differences in immune activation (13).

### *Cn* infection causes distinct glycolytic and respiratory response in BMDM

Considering the differential influence of active or VBNC *Cn* on BMDM mitochondrial ΔΨm, and that VBNC caused no detectable changes in the ΔΨm via flow cytometry, we wondered if VBNC could cause more subtle changes in BMDM cellular metabolism. To explore this, we analyzed the cell energy metabolism of infected M0-BMDM via respirometry, which measures oxygen consumption and extracellular acidification, followed by the administration of mitochondrial inhibitors to provide detailed information on mitochondrial functionality. After prior titration of host cell density and the concentration of mitochondria-modulating drugs in murine M0-BMDM, we performed respirometry using Seahorse and Oroboros equipment (23, 24). Seahorse provides insights on BMDM metabolism in the context of adhered cells, more representative of the natural condition of infected BMDM, while Oroboros allows analysis of cells in suspension, with drug concentration modulation in real time.

**Fig 2:**
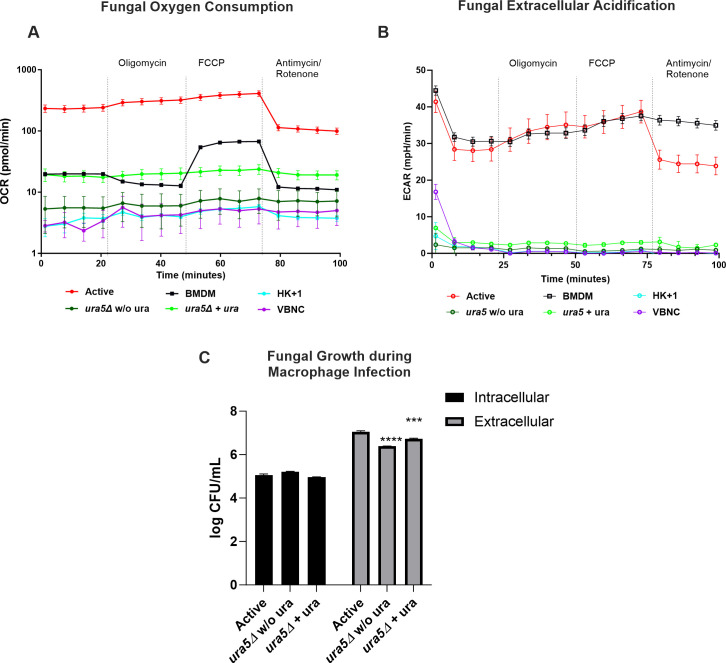
Active Wild Type (WT) *Cn* camouflages measurements of BMDM oxygen consumption. (**A**) Mitochondrial OCR of BMDM and fungal OCR of active WT (active), VBNC, HK + 1% active (HK + 1) or active *ura5*Δ (*ura5*Δ). (**B**) ECAR of BMDM vs fungal ECAR (active, VBNC, HK + 1, *ura5*Δ). (**C**) Quantification of intracellular and extracellular fungal colony forming units (CFU) from WT or *ura5*Δ active *Cn* after BMDM infection (multiplicity of infection 1:3) for 24 h. Two-way analysis of variance, where ***P < 0.001 and ****P < 0.0001 compared to active *Cn*. Figure shows the mean and SEM of one representative experiment of two independent experiments with 8–12 replicates per group.

First, we observed that VBNC and HK + 1 *Cn* fungus alone showed negligible changes in the oxygen consumption rate (OCR) and in the extracellular acidification rate (ECAR) (Fig. 2; Fig. S1B, D, and E). Because fungal protein extraction yielded undetectable protein amounts, normalization for protein was not possible, and we used raw data for analyses (Fig. 2; Fig. S1B). Consequently, oxygen consumption and media acidification from these *Cn* forms contributed negligibly to overall respirometry measurements, allowing us to directly determine signals from BMDM infected with VBNC and HK + 1 *Cn* (Fig. 2A). In contrast, active *Cn* exhibited high OCR and ECAR, interfering with measurements in infected macrophages (Fig. 2A and B). Notably, *Cn* did not respond to oligomycin and Carbonylcyanide 4-(trifluoromethoxy)phenylhydrazon (FCCP) at the concentrations used for BMDM, as their OCR and ECAR remained unchanged after administering these mitochondrial inhibitors (Fig. 2A and B).

A strategy we devised to analyze the influence of an active *Cn* on macrophage OCR was to use a *ura5*Δ strain, which is auxotrophic for uracil. We reasoned that by removing uracil, we could drastically reduce or arrest the metabolic activity of *ura5*Δ, removing fungal contribution to OCR and ECAR and thus allowing accurate measurement of macrophage metabolism. We then cultured infected macrophages in uracil-supplemented medium for 24 h, replacing it with uracil-free media 1 h before and during extracellular flux analysis. This strategy effectively reduced the baseline OCR of extracellular *ura5*Δ to more than 10-fold lower than Wild Type (WT) *Cn,* making it comparable to uninfected BMDM at the cellular density we optimized (Fig. 2A and B). To further assess that infection of BMDM with *ura5*Δ was similar to WT *Cn*, we observed if the intracellular growth of both fungi during macrophage infection was equivalent in the presence of uracil (Fig. 2C). We did not see significant differences between the intracellular growth of active WT and *ura5*Δ. Still, the *ura5*Δ strain showed significantly decreased extracellular proliferation compared to active *Cn* (Fig. 2C; Fig. S1C), further minimizing interference in extracellular flux analysis. Additionally, *ura5*Δ and WT *Cn* numbers at the time of extracellular flux analysis in the Roswell Park Memorial Institute (RPMI) medium (without BMDM) were at least fivefold higher than when infecting BMDM (Fig. S1C).

To ensure we were analyzing the OCR and ECAR of *Cn*-BMDM interaction and not just the sum of values for *Cn* and BMDM, we arithmetically summed OCR and ECAR values of VBNC and *ura5*Δ *Cn* alone with BMDM values and compared them to the OCR/ECAR of infected BMDM (Fig. S1B). This showed that the summed OCR of active *ura5*Δ (growing in wells without BMDM) + BMDM (uninfected) was not identical to the OCR of BMDM infected with *ura5*Δ (Fig. S1B). In addition, we noted that VBNC increased the OCR and ECAR in M0-BMDM cells despite negligible OCR of VBNC when cultured in isolation (Fig. S1B). Additionally, since *ura5*Δ respiration was resistant to the concentrations of oligomycin used, respiration of fungus would be detected in the non-mitochondrial OCR component of respirometry, providing an internal control. Overall, these results indicate that the contribution of *ura5*Δ to OCR and ECAR in infected BMDM is sufficiently small to give insights into BMDM immunometabolic responses, which we confirmed through subsequent analysis.

**Fig 3:**
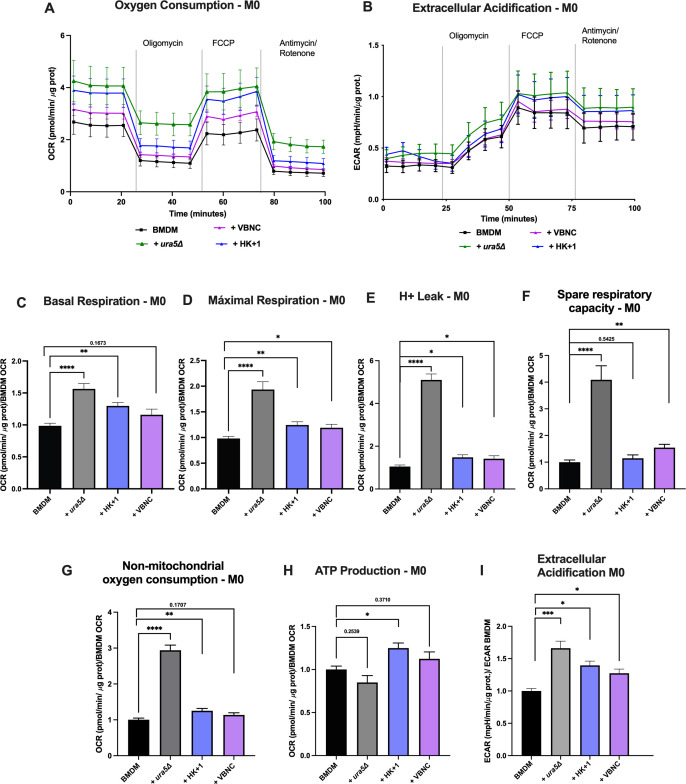
*Cn* infection increases aerobic respiration and glycolysis, and VBNC *Cn* increases maximal respiration of infected macrophages. (**A**) OCR and (**B**) ECAR of BMDM infected or not with active WT (active), active *ura5*Δ (*ura5*Δ), HK + 1% active (HK + 1), and VBNC *Cn* (multiplicity of infection [MOI] 1:3) for 24 h. Strain *ura5*Δ was supplemented with 50 μg/mL uracil during infection and removed just before analysis in Seahorse Mito Stress Test. OCR and ECAR were normalized to total protein content. Figure is representative of five independent experiments. (**C**) M0-BMDM basal respiration, (**D**) M0-BMDM maximal respiration, (**E**) M0-BMDM proton (H+) leak, (**F**) M0-BMDM spare respiratory capacity, (**G**) M0-BMDM non-mitochondrial oxygen consumption, (**H**) M0-BMDM ATP production, and (**I**) M0-BMDM ECAR infected or not with active *ura5*Δ (*ura5*Δ), HK +1% active (HK + 1) and VBNC *Cn* (MOI 1:3) for 24 h. Metabolic parameters were calculated from the OCR values detected on five independent experiments with 8–12 experimental replicates in the Seahorse Mito Stress Test divided by BMDM OCR. OCR and ECAR were normalized by protein content. Shown are mean and SEM. One-way analysis of variance statistical analysis, where *P < 0.05, ***P < 0.001, and ****P < 0.0001.

We noted that M0-BMDM infected with *ura5*Δ and with HK + 1 showed an increment in baseline OCR, while BMDM infected with VBNC showed a trend toward increased baseline OCR (Fig. 3A and C; Fig. S1E), indicating an increase in mitochondrial respiration during *Cn* infections. All fungal forms changed the maximal respiration and proton leak, with the infection with active *ura5*Δ *Cn* inducing the most pronounced effects (Fig. 3D and E). In contrast, VBNC and HK + 1 induced only a slight increase in proton leak-driven respiration, and VBNC caused a modest elevation in spare respiratory capacity (Fig. 3F). HK + 1 was the only fungal form to increase ATP-linked respiration (Fig. 3H; Fig. S1E). Infection with active ura5Δ induced higher non-mitochondrial respiration (Fig. 3G), likely representing residual fungal respiration (Fig. 2A). Furthermore, all fungal infections significantly increased ECAR in M0-BMDM (Fig. 3B and I), indicating an increase in glycolytic metabolism (22, 23). Overall, infection with VBNC caused minor changes in BMDM OCR and ECAR compared to infection with active *ura5*Δ, suggesting that these parameters are only strongly regulated after infection with the active fungal form. In the future, it would be interesting to study the *in vivo* relevance of the minor (but significant) OCR and ECAR adaptations detected when ingesting a dormant form of *Cn*.

### *Cn* modulates glucose and fatty acid metabolism in infected macrophages

Considering the increase in OCR and ECAR in M0-like BMDM infected with *Cn*, we analyzed how the fungi modulated the metabolism of glucose, glutamine, and fatty acids in M0-BMDM. The Seahorse Mito Fuel Kit (Agilent) measures this relative usage by tracking changes in OCR following the inhibition of mitochondrial import of each fuel type. A different combination of drugs provides insights into two parameters: dependency, defined as the portion of OCR driven by a specific fuel that cannot be compensated by others, and capacity, defined as the ability to use a particular fuel when others are restricted.

**Fig 4:**
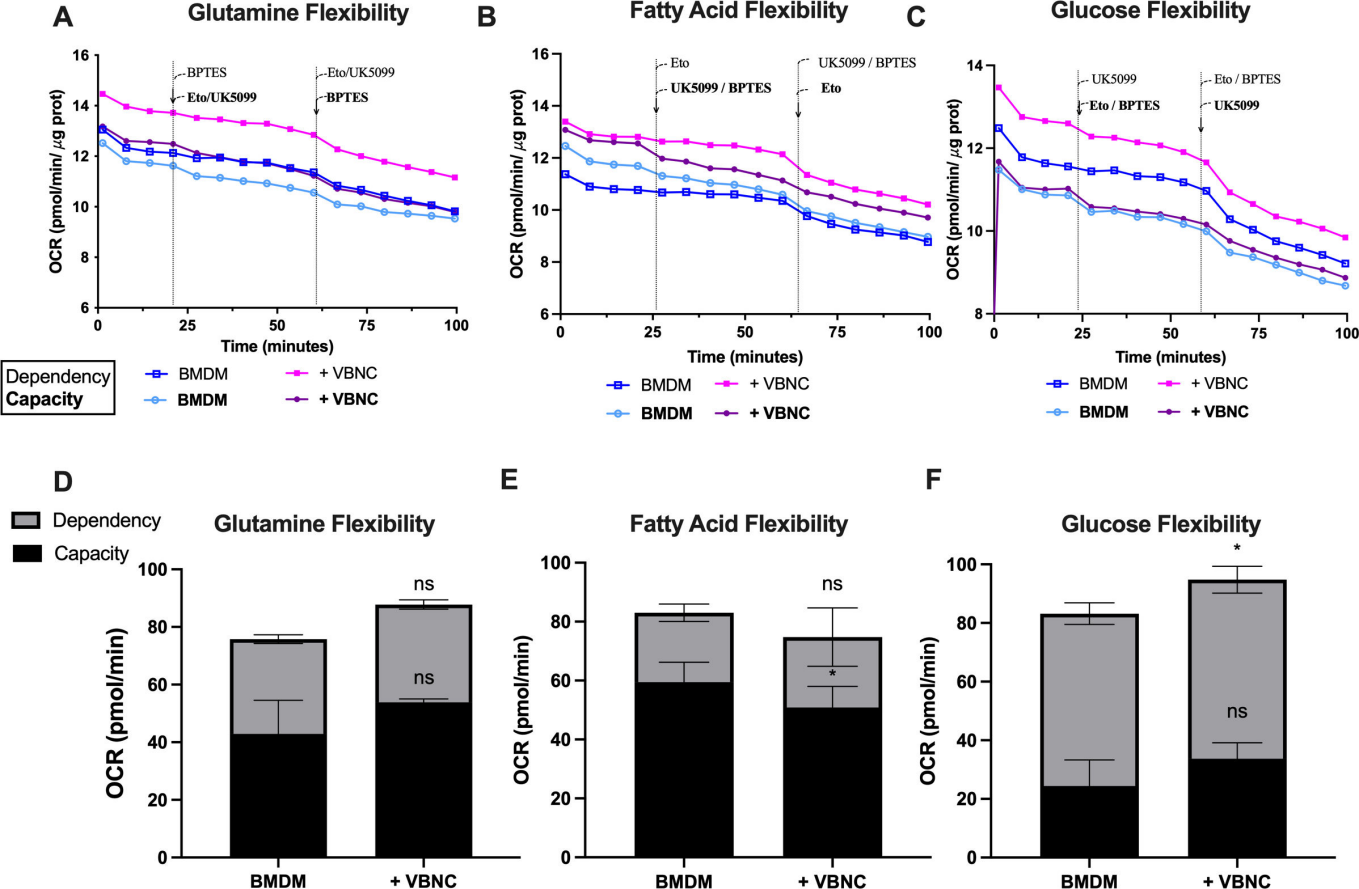
VBNC *Cn* infection of BMDM increases glucose dependency and reduces the capacity to use fatty acids. (**A–C**) OCR of BMDM after infection with VBNC *Cn* for 24 h (multiplicity of infection 1:3) after Seahorse Mito Fuel Test, with metabolism inhibitors 2 μM UK5099, 4 μM etomoxir and 3 μM BPTES, inhibitors of glucose, fatty acid, and glutamine metabolisms, respectively. Bold text indicates that those inhibitors were applied to measure capacity, and regular text indicates that those inhibitors were applied to measure dependency. (**D–F**) Capacity and dependency of utilization of (**D**) glutamine, (**E**) fatty acids, or (**F**) glucose as fuel for mitochondrial metabolism. OCR was normalized by protein content. One-way analysis of variance, where *P < 0.05 and ****P < 0.0001 compared to BMDM. Mean and SEM of a representative experiment from two independent biological replicates, each with seven experimental replicates.

To avoid interference from the residual OCR of active *ura5*Δ, we focused this analysis on VBNC *Cn*. We observed that uninfected M0-BMDM can use glucose, glutamine, and fatty acids (Fig. 4). M0-BMDM infected with VBNC showed no changes in the use of glutamine (Fig. 4A and D) but demonstrated an increased dependency on glucose (Fig. 4C and F) and decreased capacity to use fatty acids (Fig. 4B and E). This shift toward a preference for glucose over fatty acid during VBNC infection is consistent with the observed increase in ECAR (Fig. 3B and I).

**Fig 5:**
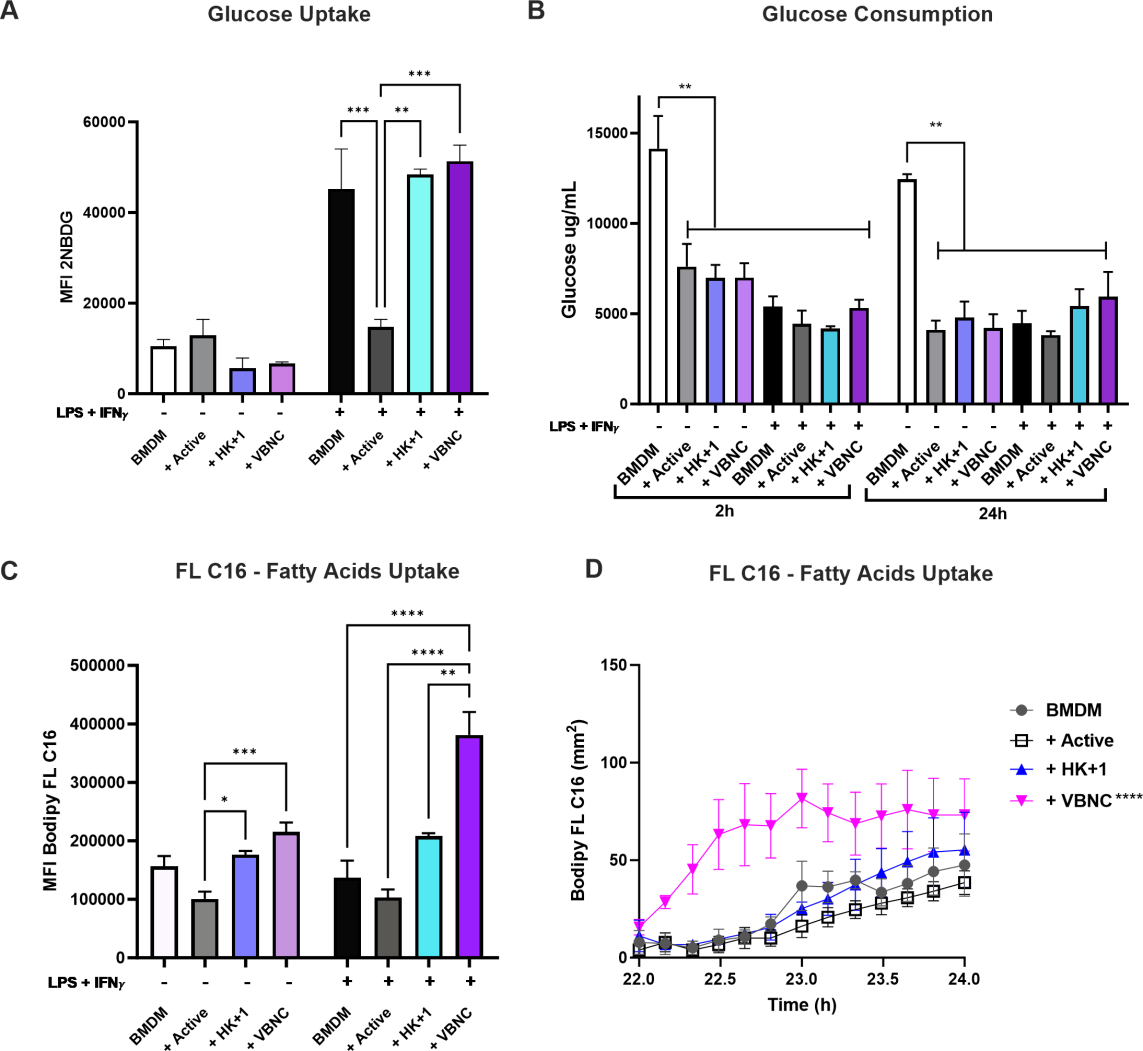
*Cn* infection of BMDM modulates glucose and fatty acid metabolism at the transcriptional level. M0-BMDM and M1-BMDM were infected with active, HK + 1% active (HK + 1), and VBNC *Cn* (multiplicity of infection 1:3) for 24 h. (**A**) MFI of 2-NBDG, used to measure glucose uptake. (**B**) Glucose concentration in extracellular medium 2 h and 24 h after infection detected by Amplex Red Glucose Assay Kit. (**C**) MFI of Bodipy FL C16, representing uptake of free palmitate. (**D**) Quantification of Bodipy FL C16 fluorescent spots in millimeters in BMDM. Spot areas were calculated from images generated by the CellCyte X microscope (Cytena) at 10× magnification and calculated by the CellCyte Studio software. Mean and SEM of a representative experiment of two independent biological replicates containing three experimental replicates. One-way analysis of variance, where *P < 0.05, **P < 0.01, ***P < 0.001, and ****P < 0.0001.

To continue the investigation of fuel usage, we then measured glucose and fatty acids uptake by infected M0- and M1-BMDM. We used a fluorescent glucose analog 2-(N-(7-nitrobenz-2-oxa-1,3-diazol-4-yl)amino)-2-deoxyglucose (2-NBDG) and labeled free palmitate (Bodipy FL C16) to track the uptake of glucose and free fatty acids, respectively (Fig. 5). In M0-BMDM infected with all forms of *Cn*, changes in 2-NBDG uptake were not detected (Fig. 5A). As previously described, M1-BMDM (14) shows a significant increase in 2-NBDG uptake (Fig. 5A). This increase was unaffected by infection with HK + 1 or VBNC (Fig. 5A). Notably, in M1-BMDM infected with active *Cn*, we observed a decrease in 2-NBDG uptake, suggesting either preference for other fuel or metabolic stress driven by impaired glucose utilization (25). We also analyzed glucose consumption by measuring glucose concentration on the extracellular supernatant after infection. We noted a decrease in glucose concentration in infected BMDM to uninfected (Fig. 5B). The glucose decrease in extracellular media is likely due to extracellular yeast consuming this glucose, perhaps analogous to *Candida albicans’* glucose consumption when infecting tissues (26).

In contrast, Bodipy FL C16 uptake was significantly reduced in M0-BMDM infected with active *Cn* compared to HK + 1 and VBNC-infected BMDM, with VBNC-infected BMDMs showing a trend toward higher palmitate uptake. In addition, fatty acid uptake did not change in M1- and M0-BMDM (Fig. 5C), as shown before (27). Interestingly, VBNC infection of M1-BMDM leads to a marked increase in palmitate uptake (Fig. 5C). We confirmed these results by fluorescent micrography of M1-BMDM from 22 to 24 h of infection. Bodipy FL C16 accumulation into visible puncta was more significant in VBNC-infected BMDM than in non-infected BMDM (Fig. 5D). These visible puncta are consistent with increased lipid droplets observed upon BMDM infection with active *Cn* (C. Coellho, unpublished data). However, we did not confirm lipid droplet formation in BMDM infected with VBNC. More work is required to understand why the uptake of free palmitate (and potentially lipid droplet formation) is higher in M1-BMDM infected with VBNC, a major metabolic difference from BMDM infected with active *Cn*. Given that VBNC infection did not significantly increase fatty acid-dependent OCR, we posit that the elevated palmitate uptake is not directed to fueling mitochondrial respiration (see Fig. 4E). Instead, it may be diverted to other metabolic pathways such as membrane remodeling, prostaglandin biosynthesis, and/or accumulation in lipid bodies (18, 28–30).

### VBNC *Cn* modulates gene expression of important glucose and fatty acid metabolism pathways in infected macrophages

**Fig 6:**
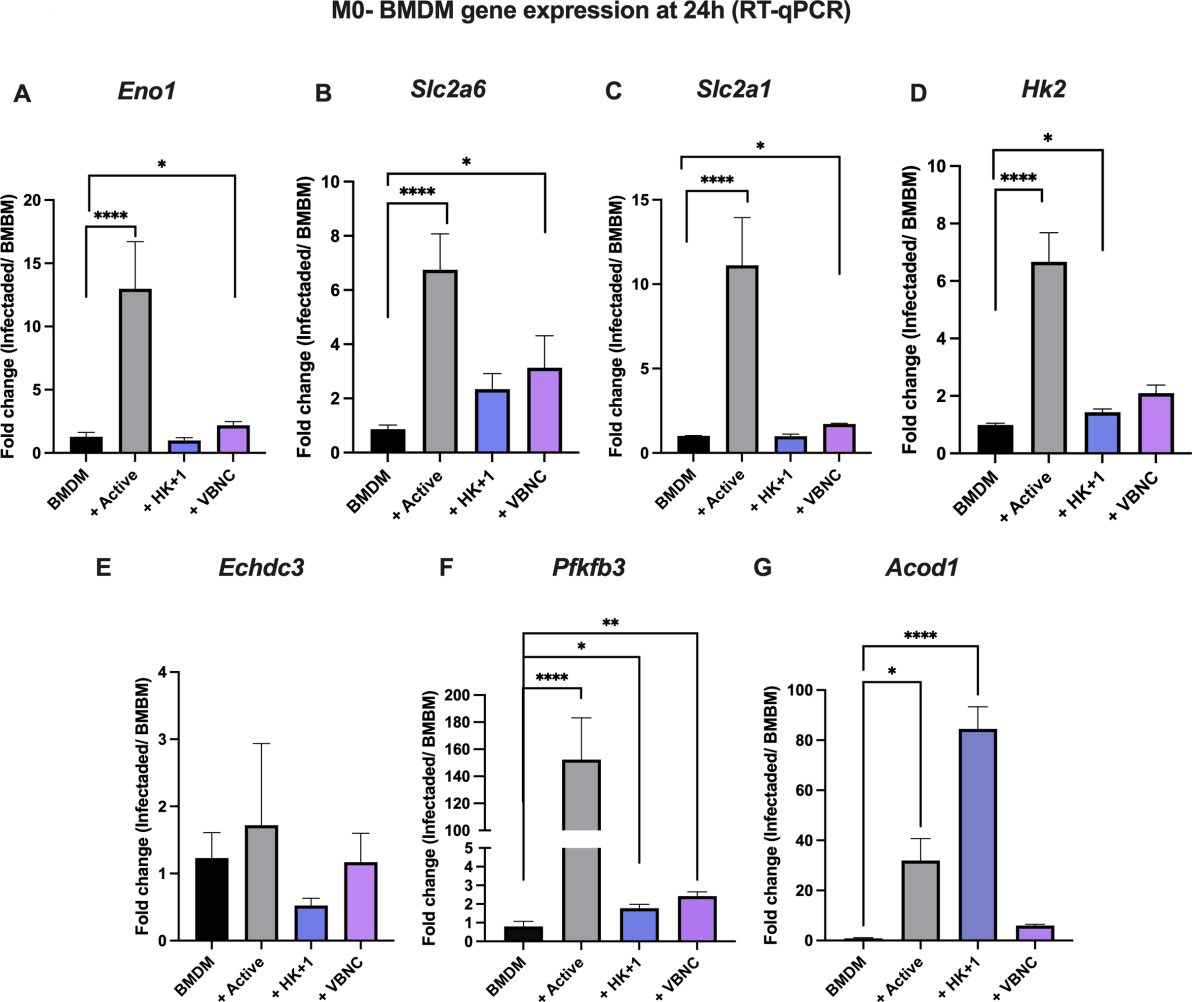
*Cn* increases expression of glycolysis-related genes (**A**) *Eno1*, (**B**) *Slc2a6*, (**C**) *Slc2a1*, (**D**) *Hk2*, (**E**) *Echdc3*, (**F**) *Pfkfb3* and (**G**) *Acod1* in infected macrophages. Fold gene expression change by RT-qPCR of M0-BMDM (untreated) infected with active, HK + 1% active (HK + 1), and VBNC *Cn* at multiplicity of infection of 3 for 24 h compared to uninfected BMDM. One-way analysis of variance, where *P < 0.05, **P < 0.01, and ****P < 0.0001. Shown are mean and SEM data from three independent experiments containing three experimental replicates.

To test whether these metabolic alterations were reflected at the transcriptional level, we selected genes encoding for metabolic enzymes which were reported before to be over-expressed upon fungal infection (24): hexokinase 2 (*Hk2*), the rate-limiting and first step of glycolysis in the cytosol; enolase1 (*Eno1*), present in the last steps of glycolysis, converting 2-phospho-D-glycerate to phosphoenolpyruvate; solute carrier family 2, member 1 (*Slc2a1*), encoding for GLUT1 in transmembrane glucose transport; solute carrier family 2, member 6 (S*lc2a6*), encoding for GLUT6, a glucose transporter with high expression in spleen and brain; enoyl coenzyme A hydratase 3-containing domain (*Echdc3*), involved in fatty acid metabolism and cellular response to insulin stimuli; 6-phosphofructo-2-kinase/fructose-2,6-bisphosphatase 3 (*Pfkfb3*), involved in the synthesis and degradation of fructose-2,6-bisphosphate and aconitate decarboxylase 1 (*Acod1*), which catalyzes the production of itaconate, a potent immunometabolic transducer. Active and VBNC infection of M0-BMDM induced a strong expression of these glycolytic genes at 24 h post-infection (Fig. 6), and infection with HK + 1 induced the expression of *Hk2, Pfkfb3,* and *Acod1* (Fig. 6). This gene expression pattern supports an increase in glycolysis during infection with *Cn* in line with the extracellular flux analysis (Fig. 3 and 4), albeit not accompanied by a strong increase in the uptake of 2-NBDG (Fig. 5A).

**Fig 7:**
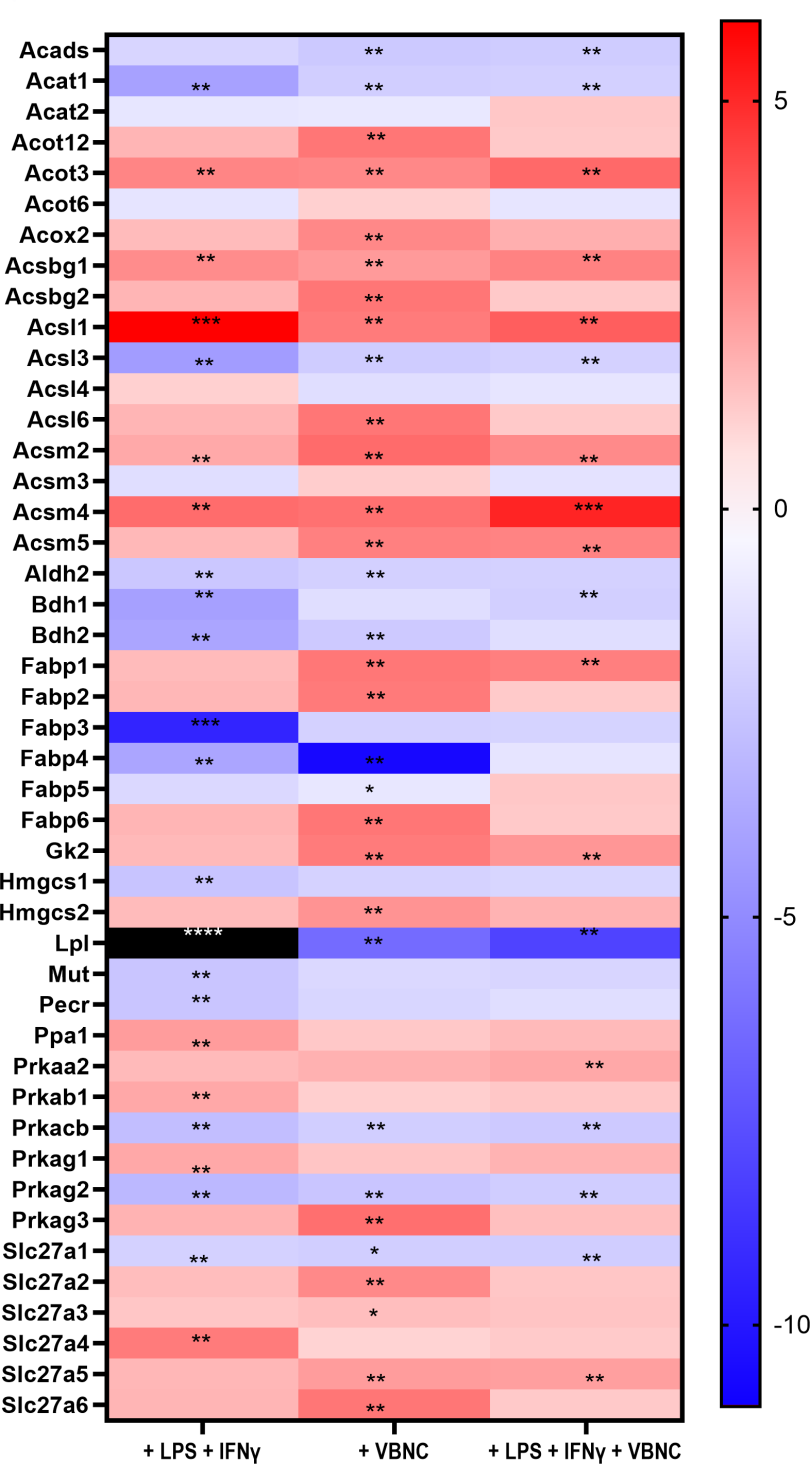
Macrophages infected with *Cn* modulate the expression of genes related to fatty acid metabolism. Gene expression of M1-BMDM (pre-treated with LPS and IFN-γ) infected with VBNC *Cn* at multiplicity of infection of 3 for 24 h, compared to M0-BMDM. The black square represents data outside the defined range, with a fold change of −56. Two-way analysis of variance, where *P < 0.05 and ****P < 0.0001 compared to uninfected M0-BMDM. Data from three independent experiments containing three experimental replicates.

**Fig 8:**
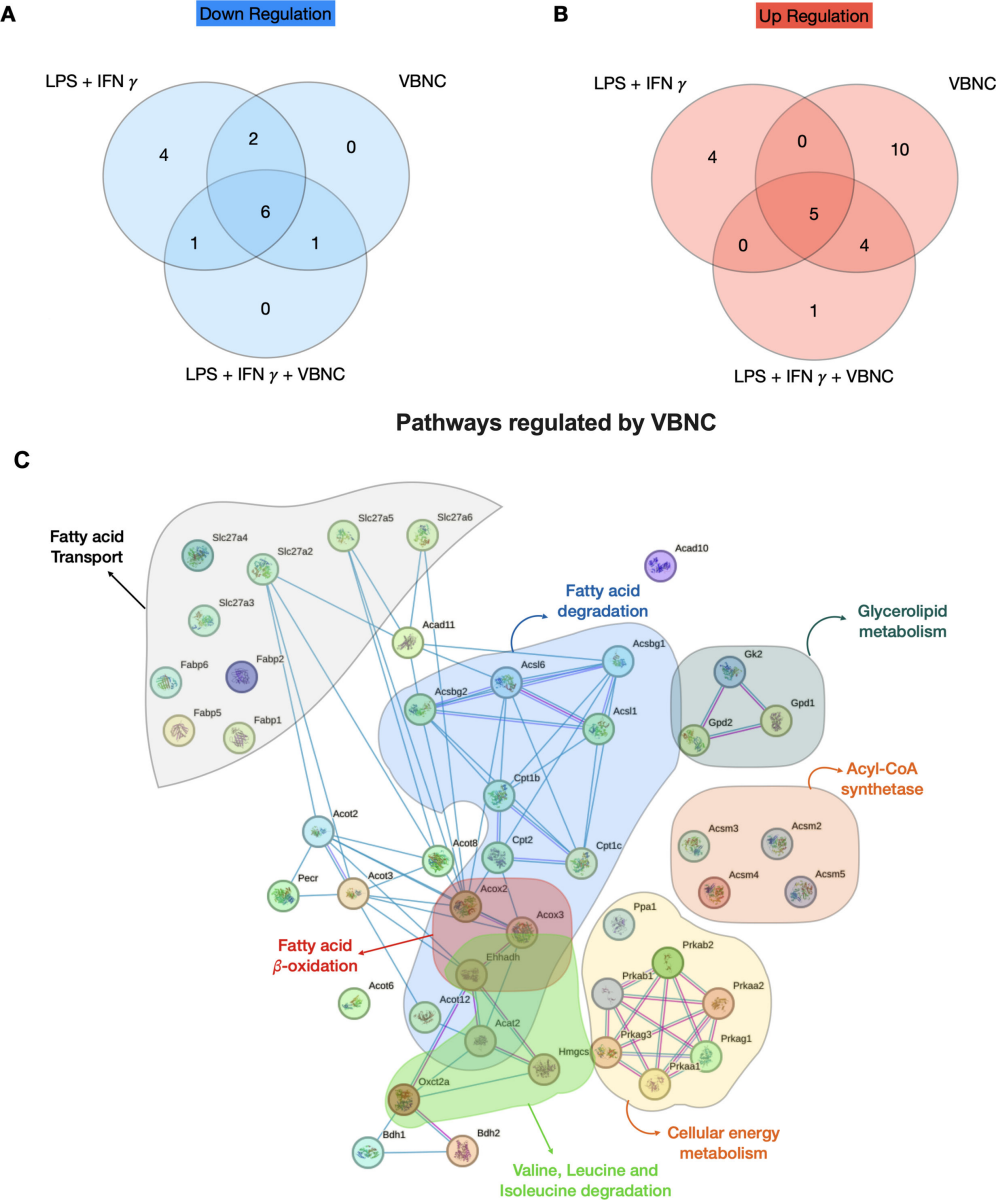
VBNC *Cn* infection induces a specific profile of fatty acid metabolism genes. (**A and B**) Venn diagram of genes differentially regulated (*P* < 0.05) down or up-regulated LPS and IFN-γ and infection with VBNC *Cn* at multiplicity of infection of 1:3 for 24 h based on fold regulation of fatty acid RT2 Profiler PCR Array concerning uninfected M0-BMDM. (**C**) Schematic representation of fatty acid gene pathways regulated by VBNC.

Given the increase in lipid uptake induced by VBNC on M1-BMDM (Fig. 5C), we analyzed the expression of important fatty acid metabolism genes using RT-qPCR array (Qiagen) in M0 and M1-BMDM infected with VBNC for 24 h (Fig. 7). As observed before (19), M1-BMDM down-regulated the expression of 14 fatty acid metabolism genes, particularly fatty acid transport and degradation. Of these 14 genes, 9 were also downregulated by *Cn* infection (Fig. 7 and 8B). Compared to M0, VBNC differentially induced the expression of 10 genes related to fatty acid transport (*Fabp2, Fabp6, Slc27a2, Slc27a6*), fatty acid β-oxidation (*Acox 2*), acyl-CoA synthase (*Acsl6*), and fatty acid degradation (*Acot12*, *Acsbg2*). M1-like BMDM infected with VBNC (Fig. 7 and 8B) also upregulates this pathway (nine genes). We detected no genes significantly down-regulated only by VBNC (Fig. 7 and 8A). In M1-BMDM, acyl-CoA synthase related genes (*Acs1*, *Acsm2*, *Acsm4*) were increased, likely associated with the previously observed fatty acid crystal formation in LPS-treated macrophages for 16 h (31). In agreement with this notion, we observed a strong downregulation of *Lpl* in all groups, with a lower downregulation in VBNC-infected BMDM (Fig. 7). *Lpl* encodes for lipoprotein lipase, the ligand/binding factor for receptor-mediated lipoprotein uptake, and it is the rate-limiting enzyme for converting circulating triglycerides to free fatty acids (32, 33). An increase in *Lpl* is a hallmark of M2 macrophages; thus, downregulation of *Lpl* is consistent with the M1 polarization induced (34), although this is attenuated by VBNC *Cn* infection.

In addition, VBNC-BMDM infection up-regulated *Fabp1* and *Fabp2* and down-regulated *Fabp4* (Fig. 7). In humans, these transporters differ in tissue expression (34), stoichiometry, affinity, and specificity toward ligands and can differentially regulate the function of immune cells, including dendritic cells (35). Notably, after 24 h of infection, *Fabp1*, the transporter of long-chain fatty acids (13–21 carbons in the aliphatic chain), was strongly up-regulated in M0 and M1-like BMDM infected with VBNC, but not by uninfected M1-BMDM. Overall, our data indicate a specific shift in lipid metabolism in BMDM induced by active and VBNC *Cn* infection, with some significant immunometabolic differences compared to active *Cn* infection at the level of lipid metabolism. Future work will decipher the role of these molecules in preventing the reactivation of *Cn* (13).

## DISCUSSION

Here, we show that metabolic adaptations during *in vitro* infection of murine BMDM with *Cn* are sensitive to different fungal growth stages and viability, in which VBNC *Cn* infection causes metabolic adaptations to a lesser degree than active *Cn* infection while causing specific differences in lipid metabolism in infected phagocytes (13). This behavior aligns with the fact that VBNC has significantly reduced protein secretion and decreased remodeling of the cell walls and fungal cellular metabolism, presumably leading to reduced capacity to manipulate host cells, usurp nutrients, and/or cause cellular damage (36). Metabolic measurements were made more difficult by the coexistence of two organisms (23), which respond to mitochondrial inhibitors differently (37).

Depending on the stimulus macrophages receive, their metabolism adapts to fuel inflammatory responses (38). Treatment with LPS and IFN-γ induces M1 profile in macrophages, characterized by a high glucose consumption rate and high glycolytic status. This is accompanied by suppressing mitochondrial oxidative phosphorylation via blockage of the citric acid cycle with accumulation of citrate, which is converted into malonyl-CoA to fuel *de novo* synthesis of fatty acids, enabling plasma membrane remodeling and organelle synthesis (14). In contrast, treatment with interleukin (IL)-4 induces M2-macrophages, characterized by increased usage of fatty acids to fuel oxidative phosphorylation (14, 39–43). M2 polarization is directly antagonized by fatty acid transport protein 1 (FATP-1) and *Fatp1* deletion favors the M1-like phenotype, including increased expression of IL-1β, IL-6, and nucleotide-binding domain, leucine-rich–containing family, pyrin domain–containing-3 (NLRP3)-related genes and glucose consumption in BMDM (44).

The composition of fatty acid in macrophage membrane phospholipids influences macrophage polarization, in which increased proportions of palmitic and palmitoleic acids favor M1. In contrast, stearic acid and n-3 polyunsaturated fatty acids favor the M2 phenotype (39). Lipid metabolism and lipid droplets also play an important role in macrophage inflammatory activity. Pro-inflammatory stimuli and intracellular infections induce the formation of lipid droplets in the cytoplasm of foamy macrophages, accompanied by mitochondrial depolarization (40, 41).

Our previous findings showed macrophage mitochondrial fission during infection with *Cn,* with no detectable damage to mitochondria and no loss of mitochondrial mass during infection (21). Instead, active *Cn* causes mitochondrial depolarization in infected macrophages mediated by NO (21), presumably because of NO’s competitive inhibition of respiratory complex IV and S-nitrosation of respiratory complex I (37, 45). In this work, we observed that infection by *Cn* caused mitochondrial depolarization accompanied by increased OCR and proton leak, indicating a specific adaptation of mitochondrial complexes, likely decoupling of super-complexes. Similarly, Garaude et al. showed transient decoupling of super-complexes of BMDM upon bacterial infection (46). This suggests that infection with *Cn* leads to increased mitochondrial electron leakage or increased uncoupling activity, which would be in line with mitochondrial fission observed previously by our group, maybe due to increased activity of uncoupling proteins, such as UCP2 (21). These adaptations are very context-specific, as depolarization not accompanied by an increase in OCR after inflammatory stimuli can be observed in BMDM infected with *Escherichia coli* (46) and after 6 h stimulation with prostaglandin E2 (PGE2) (47).

The upstream causes of mitochondrial depolarization by active *Cn* remain unknown, and an inflammatory signal that induces mitochondrial depolarization is PGE2. Production of PGE2 reduced mitochondrial ΔΨm in M0 and M2-BMDM and in CD8^+^ T cells. PGE2-induced depolarization triggers downstream cascades driven by *Slc25a11* and *Got1* with no loss of mitochondrial mass and cellular viability (47). Interestingly, *Cn* can produce authentic PGE2, which promotes fungal survival and proliferation in infected J774 murine macrophages and within zebrafish (48–50), but the immunometabolic effects of fungal-derived PGE2 are still unknown. We posit that PGE2 secretion by the fungus may contribute to mitochondrial depolarization, in addition to NO.

Lev et al. observed that *Cn* induced an increment in OCR and ECAR in infected human monocytes (23). This simultaneous increase in OCR and ECAR was also observed in response to HK *C. albicans* yeast (51), suggesting yeast intracellular residency induces a similar rise in glycolytic and respiratory responses, likely to fuel phagocytosis and inflammatory responses. Mitochondrial uncoupling could be caused by adenosine monophosphate-activated protein kinase (AMPK) activation (52), and it has been shown before that active *Cn* differentially induces phosphorylation of mTOR, STK11/LKB1, and AMPK-AIC signaling networks in infected BMDM (53). In addition, we observed before (13) that ERK1/2 pathways are modulated in BMDM during *Cn* infection, and increased ERK1/2 may contribute to regulating the glycolysis/respiration balance (54). The mixed immunometabolic profile we observed with active *Cn,* previously reported for other yeasts, is likely regulated by signal integration from several regulatory cascades in response to secreted fungal factors, nutrient competition, and pattern recognition receptor (PRR)-derived inflammatory signaling.

Here, we observed that VBNC *Cn* did not cause detectable macrophage mitochondrial depolarization, and glycolytic gene expression was attenuated in contrast with infection with active *Cn*. Our previous work showed that after 6 h of infection, transcriptional changes did not indicate a robust glycolytic profile, indicating that, during VBNC reactivation, macrophage metabolism takes several hours to switch on a glycolic response (13, 26). These observations support the notion that active growing fungi have higher secretion of immunomodulatory molecules, higher nutrient hijacking, increased stress to host cell membranes due to enlarged phagosomes, and, finally, stronger triggering of PRR/PAMP.

Strikingly, we also observed that a major difference from VBNC to active *Cn* is the significant changes in palmitate uptake and genes related to the lipid metabolism of infected macrophages. This aligns with our previous data showing that 6 h of VBNC infection, but not active *Cn* infection, induced the expression of triglyceride sequestration genes and NO synthesis in infected macrophages (13). Our data indicate that the formation of lipid aggregates in M1-BMDM is not linked to mitochondrial fuel usage since mitochondrial fatty acid oxidation is reduced in these cells (38, 41). Instead, palmitate uptake is directed toward lipid bodies and other inflammatory processes. Previous works have shown that lipid bodies are sites of production of inflammatory mediators, such as eicosanoids and prostaglandins, such as PGE2, from arachidonic acid in the lipid substrate (38, 40, 55).

Interestingly, our findings are in line with work in human bronchoalveolar lavage phagocytes *ex vivo,* in which infection with *Cn* for 2 h stimulated the expression of lipid catabolism genes in infected macrophages, mainly *Fabp4*, accompanied by greater production of tumor necrosis factor (TNF)-α, and IL-1β associated with anti-cryptococcal activity (56, 57). Strikingly, the expression of *FABP4* is also detected in cerebrospinal fluid (CSF) of AIDS-associated cryptococcosis patients (58). Thus, *Fabp4* induces cholesterol and triacylglycerol accumulation in human Tohoku Hospital Pediatrics (THP)-1 cells (59), and it is positively associated with efficient fungal killing, although these mechanisms are not fully understood. Besides that, FABP-4 was associated with protecting against *Pseudomonas aeruginosa* in mouse lung infection (60) and against *Bacillus Calmette-Guerin* in RAW264.7 cells (61).

The accumulation of fatty acids in macrophage cytoplasm is observed in infection with VBNC *Mycobacterium tuberculosis*, which is hypothesized to favor pathogen growth, allowing the hijacking of fatty acids to fuel mycobacteria growth (62). Lung granulomas are sites where dormant *M. tuberculosis* or *Cn* are found, which simultaneously restrict microorganism proliferation and provide a niche for long-term latent infection in a nutrient-rich environment (62). *Cn* induces the formation of lipid droplets in infected mice’s macrophages and lungs, which may contribute to the formation of granulomas (63). Curiously, Lim et al. have shown that unregulated lipid metabolism leads to a higher formation of granulomas in a sarcoidosis model from a mouse model using human-derived monocytes (64).

Active *Cn* can scavenge lipids from macrophages and granulomas, favoring fungal growth and nonlytic exocytosis (65). Previous works support this notion as lipids and glycolytic metabolism in *Cn* are essential for infection, given that deleting the acetyl-CoA synthetase gene *Acs1* and deletion of critical glycolytic enzymes pyruvate kinase (*pyk1*) and hexose kinases I and II (*hxk1*/*hxk2*) leads to virulence defects in *Cn* pulmonary and brain infection (66, 67). We hypothesize that the differences found in fatty acid metabolism of BMDM infected with VBNC *Cn* are derived from the fungal metabolism, as we have shown previously that fungal fatty acid metabolism pathways are important for maintaining VBNC viability during dormancy (36). VBNC *Cn* had significantly lower global metabolic activity compared to the active fungus, and VBNC produces at least 63 proteins that are different from active *Cn*, mainly proteins integrative of fatty acid degradation pathways (36).

A major drawback of this work is that findings from metabolic profiles are very model- and context-dependent (68, 69). Our work underscores this complexity, showing that the microbial growth state can also influence host metabolism and that findings from *in vivo* work will inherently be complex. Continuing the study of *Cn* VBNC cells and the mechanisms involved in their reactivation is essential for the future development of new treatments for cryptococcosis. Future studies will focus on the detailed study of timelines of metabolic adaptations in *in vivo* and *in vitro* heterogeneity, according to different immune subset cells. This is justified, as most cases of the disease occur due to the reactivation of a latent infection that resurfaces when the organism shows suppression of its immune mechanisms. Furthermore, studying immunometabolism in the face of this infection is essential for understanding the parasite-host relationship, which could also be regulated pharmacologically to preventing disease progression.

## MATERIALS AND METHODS

### Fungal culture

The strains used in this study were *Cn* H99O (var. grubii) and a fluoroorotic acid-resistant H99 (ura5Δ) strain, auxotrophic for uracil, which is derived from H99 (70). Both were stored at −80°C with yeast extract-peptone-dextrose (YPD) medium (Sigma) and 25% vol/vol glycerol. The week before each experiment, an aliquot was plated on YPD agar and incubated for 3 days at 30°C. After 3 days, colonies were scraped and inoculated in a ventilated flask with 10 mL of liquid YPD for 22 h, 150 revolutions per minute (RPM) at 30°C. For this step, the *ura5*Δ strain was supplemented with 50 μg/mL of uracil. After 22 h, the fungus reached the stationary growth phase (active). For HK fungus, we heated 1 mL fungal suspension of 10^7^ cells/mL for 1 h at 75°C in a dry bath.

### Viable but not cultivable cells

After the fungus had grown for 22 h, in which fungi reached the early stationary active phase (active stage 1), 100 μL of active stage 1 was inoculated into each of two flasks containing 10 mL of liquid YPD, which was incubated for 20 h, 150 RPM, at 30°C. After 20 h of growth, the fungus reached stationary growth again (10^8^ cells / mL—active stage 2); we placed flasks in a hypoxic environment using the hypoxia generator (<0.1% oxygen) (GENbag anaer, Biomérieux) and incubated it for 7 days at 30°C. Fungal culturability and viability were monitored in each experiment to confirm the VBNC phenotype before proceeding to subsequent experiments (36).

For culturability, the fungus was washed with PBS, and 1 × 10^4^ cells were plated on a YPD agar plate and incubated for 3 days at 30°C. After 3 days, the colonies were counted, representing the number of cultivable yeasts in relation to the initial number inoculated. Fungal cell viability was confirmed by LIVE/DEAD Fixable Green (488 nm excitation/530 nm emission, Invitrogen) according to the manufacturer’s recommendations (1 μL / mL for 10^6^ cells for 20 min) and analyzed in a flow cytometer (Accuri C6 plus or BD FacsVerse). The ideal conditions for performing the following experiments were culturability of at most 5% of the initial inoculum, an average of 1% cultivability, and viability of at least 70%. We compared VBNC with active yeasts taken from stationary growth (active stage 2—active) in infection experiments. To decipher the specific contribution of VBNC cells to infection, compared to the same number of culturable cells, we represented 1% culturability by adding 1% active fungus to a suspension with 99% HK fungus (HK + 1).

To control the culturability and reactivation capacity of dormant yeasts, 1 × 10^4^ yeasts were incubated in 20 mL of minimal medium (MM), consisting of 15 mM dextrose, 10 mM MgSO_4_, 29.4 mM KH_2_PO_4_, 13 mM glycine, and 3 µM thiamine, with 10% fetal bovine serum (FBS) or only MM. Both were distributed in a 96-well plate (200 μL per well) and incubated for 5 days at 30°C. After 5 days, the wells with fungal growth (positive wells) were counted vs total wells, and the following formula was used to determine the culturability (C) and reactivation (R), in which *x* = positive growth wells; *y* = total wells; *Cfbs* = C on the plate with 10% FBS; and *Cmm* = C on the plate with only MM (18):


$$C=1-\frac{\left(1-xy\right)}{100}R=\frac{Csfb}{Cmm}$$

### Bone marrow-derived macrophages

To obtain BMDM, marrow from femurs and tibias from 8- to 12-week-old C57BL/6 mice were removed by rinsing with RPMI-1640 (Sigma-Aldrich). Cells were centrifuged at 300 × *g*, for 5 min, at 4°C, resuspended in red blood cell lysis buffer (Sigma) for 1 min, washed with RPMI and seeded at a density of 2 × 10^6^ cells/ 10 cm^2^ Petri dish in 10 mL of differentiation medium, comprised of RPMI supplemented with 50 μg/mL gentamicin, 20% (vol/vol) FBS, and 30% (vol/vol) L929-fibroblast conditioned medium (LCCM). The plates were kept in a 5% CO_2_ atmosphere, 37°C for 7 days, and on the 4th day, they were supplemented with an additional 10 mL of the differentiation medium. On day 7 of differentiation, cells and debris in the supernatant were discarded and adhered differentiated BMDM was stripped with TrypLE Express (Gibco). The purity of BMDMs was confirmed by flow cytometry analysis, with over 86% of the cells expressing F4/80. Then, BMDMs were washed, resuspended in RPMI with 10% FBS and 5% LCCM (experimental media), and plated at a density of 1 × 10^6^/mL, for at least 24 h at 37°C with 5% CO_2_ before stimuli or infections.

### BMDM infection with *Cn*

After 24 h of resting, BMDMs were either treated or not with 100 ng/mL of LPS (*Escherichia coli* serotype O111:B4, Sigma) and 50 ng/mL IFN-γ (Invitrogen) for 24 h at 37°C and 5% CO_2_. Then, the treatment was replaced with 500 ng/mL LPS and 50 ng/mL IFN-γ. VBNC, active, and HK + 1 *Cn* were incubated for 30 min at 37°C with 10 μg/mL of 18B7 anti-glucuronoxylomannan antibody for opsonization (71) and subsequently added to the BMDM in a ratio of 3 fungi to 1 BMDM (multiplicity of infection [MOI] 1:3) (72, 73). Infections were allowed to proceed for 24 h at 37°C with 5% CO_2_.

### Flow cytometry analyses

To analyze mitochondrial depolarization, 1 h before the end of the infection period, 400 ng/mL of TMRM perchlorate (ThermoFisher) was added to the cells. As mitochondrial staining and depolarization control, 15 minutes before the end of the experiment, carbonylcyanide-m-chlorophenylhydrazone (CCCP—50 μM) was added to some of the BMDM-containing wells. CCCP opens the permeability transition pore on the mitochondrial membrane, leading to the dissipation of mitochondrial membrane potential (ΔΨm), and almost complete depolarization. To analyze ROS production, 30 min before the end of the experiment, 5 μM of CellRox Green (Invitrogen) was added to the cells. We stained the cells with MitoTracker Green (Invitrogen—50 nM) for mitochondrial mass for 30 min.

To quantify fatty acid uptake, 1 h before the end of the experiment, the BMDM media were exchanged for media without FBS and 25 ng/mL Bodipy FL C16 (ThermoFisher). To quantify glucose uptake, the medium was exchanged for PBS with the 50 μM of glucose analog 2-NBDG (ThermoFisher). At the end of the infection period, BMDMs were washed with warm PBS and collected using ice-cold PBS supplemented with EDTA (10 mM) and lidocaine (4 mg/mL). BMDMs were then washed with ice-cold PBS and resuspended in anti-mouse CD16/CD32 (Fc Block, BD Biosciences) (2 μg/mL in PBS with 2 mM EDTA) on ice for 15 min. For gate strategy (Fig. S1A), BMDMs were stained with 40 ng/mL of anti-mouse CD45-APC (BioLegend) and Live/dead (Zombie NIR, BioLegend, 1:1,000) to identify viable macrophages via flow cytometer (Accuri C6 plus). Unstained, single-stained, and dead BMDM controls were included to inform compensation, gating, and analysis.

### Respirometry of infected BMDM

For oximetry analysis in Seahorse equipment, 7.5 × 10^4^ BMDMs were plated in each well of a 96-well XF96 plate (Agilent) with 80 μL of BMDM differentiation media and maintained at 37°C and 5% of CO_2_. After 24 h, fungi were inoculated to the BMDM at an MOI of 3 or to empty wells at the same concentration, and infection proceeded for another 24 h. One hour before the end of the infection period, BMDMs were washed with warm medium XF Dulbecco’s Modified Eagle Medium Agilent) supplemented with glucose (10 mM), glutamine (2 mM), and pyruvate (1 mM); the plate was incubated for 1 h at 37°C without CO_2_ followed by incubation in Seahorse equipment (Agilent). For Seahorse respirometry analysis, we used a Mito Stress Kit (Agilent) according to the manufacturer’s recommendations, using 1 μM oligomycin to inhibit ATP synthase, 1.5 μM of FCCP to deregulate the proton gradient and 0.5 μM of rotenone and antimycin for inhibition of complexes 1 and 3 respectively, as previously titrated. For energy metabolism analysis, we used the Mito Fuel Kit (Agilent), containing 2 μM UK5099, 4 μM etomoxir and 3 μM BPTES, inhibitors of the glucose, fatty acid and glutamine metabolisms respectively. Both Mito Stress and Mito Fuel analyses were performed in Seahorse for 100 min as the kits recommend. At the end of Seahorse analysis, plates were subjected to protein quantifications for normalization, by lysing BMDM with NaOH (1 mM), followed by protein concentration determination via Bradford solution (Bio-Rad, catalog 500-0006) according to the manufacturer’s instructions.

For oximetry analysis in the Oroboros, BMDMs were plated in Petri dishes at a density of 1 × 10^6^ cells/mL of RPMI with 10% FBS for 24 h. The next day, the cells were infected with VBNC and HK + 1 *Cn* for 24 h at 37°C and 5% CO_2_; the cells were collected using TrypLE Express, washed, resuspended in RPMI medium without FBS, and counted. OCR was measured at 37°C using 750 rpm continuous stirring in a 2 mL chamber at a density of 1 × 10^6^ cells/mL in the Oroboros Oxygraph-O2K. To inhibit mitochondrial complexes, we used oligomycin (1 μg/mL), CCCP (13 μM), rotenone (1 μM), and antimycin A (1 μM).

The calculations to determine respiration profile, fuel capacity or dependency were performed according to the manufacturer’s recommendations. For respiration profile in both Seahorse and Oroboros technology, the calculations followed Mito Stress Kit recommendation and are described in Table S1. For Mito Fuel calculations, dependency and capacity were calculated using the following formulas:


$$\begin{array}{r} Dependency=\frac{BaselineOCR-TargetinhibitorOCR}{BaselineOCR-allinhibitorsOCR}*100\\ Capacity=\left[1-\frac{BaselineOCR-Other2inhibitorsOCR}{BaselineOCR-allinhibitorsOCR}\right]*100 \end{array}$$

### Glucose consumption

BMDMs were differentiated and infected as described before, with the difference that they were washed three times with warm PBS after 2 h of infection to remove extracellular yeasts, and a new medium was added to the cells. The supernatant was collected after 2 h, 4 h, and 24 h, and glucose concentration was measured using the Amplex Red Glucose Assay Kit (Invitrogen) following the manufacturer’s instructions.

### Microscopy analysis

BMDMs were differentiated and infected as described before. After 22 h of infection, macrophages were washed three times with warm PBS to remove extracellular yeasts and a new RPMI medium was added with 25 ng/mL Bodipy FL C16 (ThermoFisher). After 30 min, we took pictures every 10 min for 2 h in a CellCyte X microscope, and analysis was performed in the CellCyte Studio.

### Gene expression

To analyze gene expression in infected macrophages, after 24 h of infection, infected and stimulated BMDMs were washed with a warm RPMI medium. RNA was extracted using an RNeasy Mini-Kit (Qiagen), following the manufacturer’s instructions. The RNA was quantified using a NanoPhotometer P330 nanophotometer (IMPLEN), and integrity was analyzed using an agarose gel. The RNA was then converted to cDNA using RT^2^ First Strand Kit (Qiagen), following the manufacturer’s recommendations. The cDNA was quantified using qRT-PCR in StepOne equipment (Applied Biosystems) using RT^2^ Sybr Green ROX qPCR Mastermix (Qiagen). We first selected some important genes for glucose metabolism based on our previous work (13, 21), as described in Table 1. Additionally, we performed RT^2^ Profiler PCR Array Mouse Fatty Acid Metabolism (Qiagen) containing 84 key genes associated with fatty acid metabolism. Data analysis was performed online using Qiagen software GeneGlobe Data Analysis Center, in which the housekeeping genes (*Actb*, *B2m,* and *Gapdh*) had constant mRNA levels between controls and experimental groups and were used for data normalization. Fold differences in transcript levels between control and experimental groups were obtained using the comparative threshold method (2^-ΔΔCt^ algorithm). Two criteria were employed to identify significantly modulated genes: (i) the fold difference in average 2^-ΔΔCt^ values was greater than 2 or less than −2 (upregulation or downregulation, respectively) and (ii) the difference of the replicate 2^-ΔΔCt^ values for each gene in the control group and experimental groups was statistically significant (*P* ≤ 0.05) according to Student’s *t*-test.

**TABLE 1:** Primer sequence used to quantify gene expression of macrophages infected with *C. neoformans* by RT-qPCR

Gene	Protein	Sequence 5´-3´
NM_Eno1_For	Enolase1	gggtggattcgcacctaac
NM_Eno1_Rev	Enolase1	atccatgccaatgacaacct
NM_slc2a6_For	Solute carrier family 2, member 6	aggatgctgacgggcttt
NM_slc2a6_Rev	Solute carrier family 2, member 6	gggtgcaatctcagacacg
NM_Slc2a1_For	Solute carrier family 2, member 1	gtcggcctctttgttaatcg
NM_Slc2a1_Rev	Solute carrier family 2, member 1	ttggagaagcccataagcac
NM_HK2_1_For	Hexoquinase 2	ttcaccttctccttcccttg
NM_HK2_1_Rev	Hexoquinase 2	ccctttgtccacttgaggag
NM_Echdc3_For	Domain containing enoyl coenzyme A hydratase 3	atacaggggagggaacgact
NM_Echdc3_Rev	Domain containing enoyl coenzyme A hydratase 3	gtatgggcagcacctcactt
NM_Pfkfb3_For	6-Phosphofructo-2-kinase/fructose-2,6-bisphosphatase 3	aacagctttgaggagcgtgt
NM_Pfkfb3_Rev	6-Phosphofructo-2-kinase/fructose-2,6-bisphosphatase 3	ccgggagctcttcatgttt
NM_Acod1_For	Aconitate decarboxylase 1	gcttttgttaatggtgttgctg
NM_Acod1_Rev	Aconitate decarboxylase 1	aagccaaagacataccaaagaga

### Statistical analysis

The figures in the present study are representative of at least three technical and biological replicates, with three independent experiments, as indicated in Figure legends. The data are represented by the media of obtained values and their standard deviation unless otherwise stated in the Figure legend. Statistical analysis was performed by one-way or two-way analysis of variance in GraphPad Prism, version 9.0, and the *P*-values represented in Figures by *P < 0.05, **P < 0.01, ***P < 0.001, and ****P < 0.0001.

## SUPPLEMENTAL MATERIAL

Fig. S1 iai.00487-24-s0001.pdf Gate strategy, fungal growth and ECAR by Oroboros.

Table S1 iai.00487-24-s0002.docx Calculation of respiration parameters for analysis performed in Seahorse and Oroboros equipments.

ASM does not own the copyrights to Supplemental Material that may be linked to, or accessed through, an article. The authors have granted ASM a non-exclusive, world-wide license to publish the Supplemental Material files. Please contact the corresponding author directly for reuse.
