# Supplementary material for: Immunometabolic reprogramming in macrophages infected with active and dormant Cryptococcus neoformans: differential modulation of respiration, glycolysis, and fatty acid utilization
Source: Infect Immun. 2024 Dec 23;93(2):e00487-24. doi: 10.1128/iai.00487-24 (PMC11834436; doi:10.1128/iai.00487-24)
Supplement: Fig. S1 — Gate strategy, fungal growth and ECAR by Oroboros. [file iai.00487-24-s0001.pdf]

A Related to figure 1 -
Gating Strategy

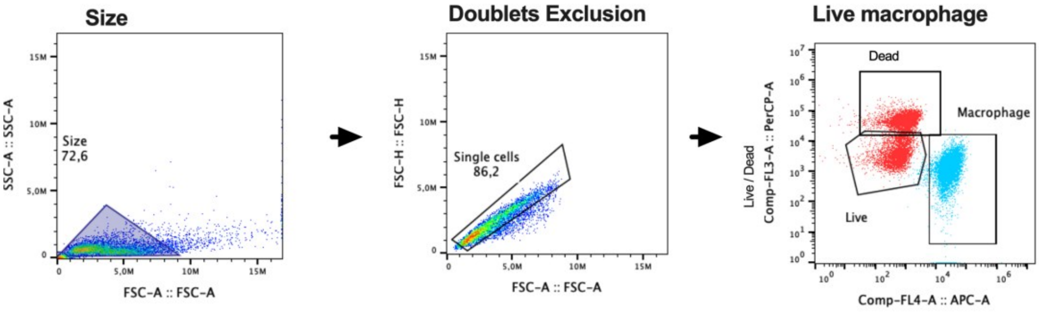

B Related to figure 2 and 3 -

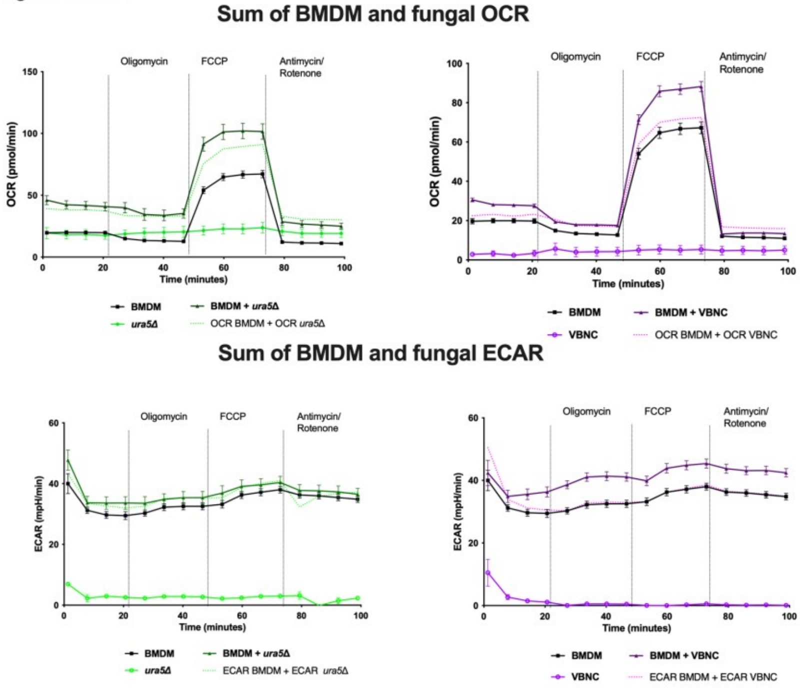

C Related to figure 2 -
Fungal Growth

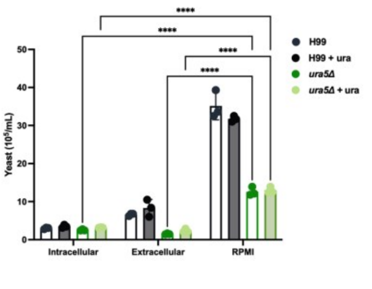

D Related to figure 3 -
Respiration Pattern in Oroboros

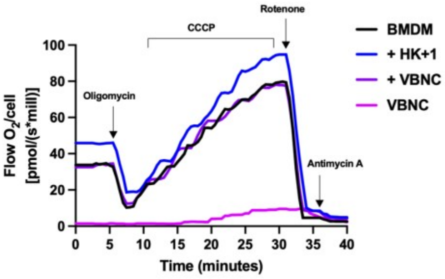

E Related to figure 3 -
Respiration Parameters in Oroboros

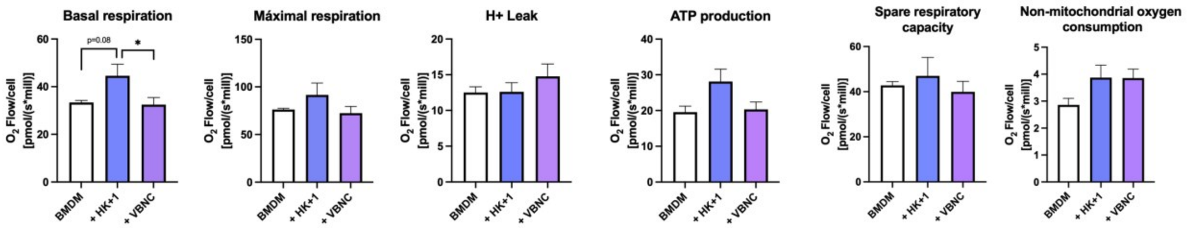

Figure S1: (A) Gating strategy for cytometry analysis. Gating strategy to identify live BMDM: CD45-APC+ (macrophages) and excluding Zombie-NIR- (Live/Dead) to analyze mitochondrial depolarization, palmitate, and glucose uptake. Figs represent single color samples: CD45-APC, and for dead/live BMDM (40C heat-shock for 2min) followed by staining with Zombie-NIR. **(B) *Cn* infection increases BMDM OCR and ECAR more than the sum of separate signals.** Mathematical sum of OCR and ECAR signals of uninfected BMDM with *ura5Δ Cn* and VBNC *Cn* (dotted line) versus real measurements of fungal and BMDM OCR or BMDM infected with *Cn* (continuous line). Fig shows one representative experiment, from a total of 5 independent experiments. **(C) WT *Cn* and *ura5Δ Cn* show similar growth inside macrophages.** Quantifying intracellular and extracellular fungal CFU of *Cn* WT H99 or *ura5Δ* after BMDM infection (MOI 1:3) for 24h. Growth in RPMI media (without infection of BMDM) in parallel wells is shown. Mean and SEM in Figs representative of three independent experiments with three experimental replicates. Two-way ANOVA ****$p < 0.0001$. **(D, E) VBNC *Cn* causes small increase changes in aerobic respiration of infected macrophages, measured in Oroboros instrument.** (**D**) Trace of Mitochondrial Oxygen (O_2) flow consumed per cell from one representative experiment (**E**) Basal respiration, Maximal respiration, H^+ leak, ATP production, Spare respiratory capacity, Non-mitochondrial oxygen consumption of BMDM infected or not with HK + 1% active (HK+1) or VBNC *Cn* (MOI 1:3) for 24h. Rates were evaluated in Oroboros equipment. One-way ANOVA *$p < 0.05$. Mean and SEM of all data from 5 independent experiments with 5 experimental replicates.
