# Supplementary material for: Immunometabolic reprogramming in macrophages infected with active and dormant Cryptococcus neoformans: differential modulation of respiration, glycolysis, and fatty acid utilization
Source: Infect Immun. 2024 Dec 23;93(2):e00487-24. doi: 10.1128/iai.00487-24 (PMC11834436; doi:10.1128/iai.00487-24)
Supplement: Table S1 — Calculation of respiration parameters for analysis performed in Seahorse and Oroboros equipments. [file iai.00487-24-s0002.docx]

Table S1. Calculation of respiration parameters for analysis performed in Seahorse and Oroboros equipments.

| Parameter Value | Equation |
| --- | --- |
| Non-mitochondrial Oxyden Consumption | Minimum rate measurement after Rotenone/antimycin A injection |
| Basal Respiration | (Last rate measurement before first injection) - (Non-Mitochondrial Respiration Rate) |
| Maximal Respiration | (Maximum rate measurement after FCCP injection) - (Non-Mitochondrial Respiration) |
| H+ (Proton) Leak | (Minimum rate measurement after Oligomycin injection) - (Non-Mitochondrial Respiration |
| ATP Production | (Last rate measurement before Oligomycin injection) - (Minimum rate measurement after Oligomycin injection) |
| Spare Respiratory Capacity | (Maximal Respiration) - (Basal Respiration) |
